# Constitutive AKT Activity Predisposes Lung Fibrosis by Regulating Macrophage, Myofibroblast and Fibrocyte Recruitment and Changes in Autophagy

**DOI:** 10.4236/abb.2019.1010027

**Published:** 2019-10-30

**Authors:** Duaa Dakhlallah, Yijie Wang, Tierra A. Bobo, Emily Ellis, Xiaokui Mo, Melissa G. Piper, Timothy D. Eubank, Clay B. Marsh

**Affiliations:** 1 Division of Pulmonary, Allergy, Critical Care & Sleep Medicine, Davis Heart and Lung Research Institute, The Ohio State University, Columbus, OH, USA; 2 Robert C. Byrd Health Sciences Center, West Virginia University, Morgantown, WV, USA; 3 The Center for Biostatistics, The Ohio State University, Columbus, OH, USA; 4 Department of Microbiology, Immunology and Cell Biology, West Virginia University, Morgantown, WV, USA

**Keywords:** Pulmonary Fibrosis, AKT, CSF1, M-CSF Receptor, Macrophage, Myofibroblast, Fibrocytes, Autophagy

## Abstract

The etiology and pathogenesis of pulmonary fibrosis is poorly understood. We and others reported that M-CSF/CSF-1, M-CSF-R and downstream AKT activation plays an important role in lung fibrosis in mice models and in IPF patients. To understand potential molecular pathways used by M-CSF-R activation to direct lung fibrosis, we used a novel transgenic mouse model that expresses a constitutively-active form of AKT, myristoylated AKT (Myr-Akt), driven by the *c-fms* (M-CSF-R) promoter. We were particularly interested in the basal immune state of the lungs of these Myr-Akt mice to assess M-CSF-R-related priming for lung fibrosis. In support of a priming effect, macrophages isolated from the lungs of unchallenged Myr-Akt mice displayed an M2-tropism, enhanced co-expression of M-CSF-R and *α*-SMA, reduced autophagy reflected by reduced expression of the key autophagy genes *Beclin*-1, MAP1-*Lc*3*a*(*Lc*3*a*), and MAP1-*Lc*3*b*(*Lc*3*b*), and increased *p*62/*STSQM*1 expression compared with littermate WT mice. Furthermore, Myr-Akt mice had more basal circulating fibrocytes than WT mice. Lastly, upon bleomycin challenge, Myr-Akt mice showed enhanced collagen deposition, increased F4/80+ and CD45+ cells, reduced autophagy genes *Beclin*-1, *Lc*3*a*, and *Lc*3*b* expression, and a shorter life-span than WT littermates. These data provide support that M-CSF-R/AKT activation may have a priming effect which can predispose lung tissue to pulmonary fibrosis.

## 1. Introduction

Idiopathic pulmonary fibrosis (IPF) is a progressive respiratory disease with a 5-year mortality rate of 50% - 70% [1]. IPF is characterized pathologically by destruction of lung architecture, fibrotic remodeling, accumulation of myofibroblasts, and deposition of extracellular matrix (ECM) [2]. To date, the etiology and pathogenesis of IPF remains unclear.

The serine/threonine protein kinase, AKT, is downstream of many receptor tyrosine kinases and plays an important role in cell survival, differentiation, and cellular activation [3]. In macrophages, activation of the M-CSF receptor (M-CSF-R) induces stimulation of phosphoinositide 3-kinase (PI3K) leading to AKT recruitment to the cell membrane and subsequent Thr308 and Ser437 phosphorylation to activate AKT [4]. In fibroblasts, activated AKT regulates collagen I and III production [5] and contributes to human hepatic fibrosis [6] and bleomycin-induced lung fibrosis in mice [7] [8]. Importantly, PI3K/AKT activity is enhanced in IPF fibroblasts due to reduced levels of PTEN phosphatase function [9]. Moreover, PI3K/AKT inhibitors such as PX-866 are potential therapeutic agents for IPF [7] [10]. Thus, understanding the role of activated AKT is important in IPF.

Macrophages express several fibrotic mediators and play an important role in wound healing and organ fibrosis [11] [12] [13] [14]. There are two main types of macrophages with disparate inflammatory functions: classically-activated “killing” macrophages (M1 phenotype) activated by microbial agents to express pro-inflammatory cytokines like TNF-*α*, IL-18, IL-12, IL-6, IL-1 and inducible nitric oxide synthase (iNOS) that enhance type I helper T cells to proliferate [15] [16] [17], and alternatively-activated, or M2 “repair” macrophages that express relatively higher levels of the anti-inflammatory cytokines IL-10, mannose receptor-1 (MRC)-1, YM1, and arginase-1 (ARG1) and Th2-type cytokines. M2 macrophages exhibit pro-fibrotic, pro-angiogenic, and anti-inflammatory activities [15] [16] [18]. Several studies suggest M2 macrophages contribute to fibrotic lung diseases like IPF [11] [19] [20]. Thus, understanding the molecular regulation of M2 macrophages may be important in pulmonary fibrosis.

Another critical effector cell type in IPF is myofibroblasts, which secrete excessive extracellular matrix proteins like collagen I and III that contribute to fibrosis [19] [21]. Myofibroblasts are identified by their expression of *α*-smooth muscle actin (*α*-SMA) and can differentiate from resident lung fibroblasts, epithelial-mesenchymal cells, or circulating fibrocytes [21] [22]. Interestingly, PI3K/AKT is involved in TGF *β*-induced myofibroblast proliferation and differentiation in IPF [23]. Thus, myofibroblast function is critical in IPF.

In addition to M2 macrophages and myofibroblasts, circulating fibrocytes are also associated with pulmonary fibrosis [24]. Fibrocytes are bone marrow-derived mesenchymal cells and express leukocyte, hematopoietic cell and fibroblast markers [25]. Fibrocytes express chemokine receptors and adhesion molecules and can traffic to fibrotic regions [24]. Furthermore, fibrocytes can deposit the ECM proteins collagen I and III [26] and can also differentiate into fibroblasts and myofibroblasts, which further contribute to ECM deposition [27]. Therefore, circulating fibrocytes can contribute to the pathogenesis of pulmonary fibrosis [24] [28].

Reduced cellular homeostatic processes like autophagy are also found in the lungs of patients with IPF [29] [30] [31]. Autophagy is an evolutionarily-conserved lysosome-dependent cellular pathway responsible for the processing of unhealthy organelles and cellular waste to maintain cellular homeostasis during cellular stress [32]. Successful autophagy results in the delivery of proteins and/or organelles into double-membrane autophagosomes for lysosomal degradation [32] [33] and cellular homeostasis. Interestingly, autophagy activity correlates with organismal longevity [34] and interruption of autophagy is associated with cellular aging. Several studies report reduced autophagic vesicle formation (autophagosomes) and activity in human IPF lung tissue as measured by reduced LC3A/B-II (microtubule associated protein 1 light chain 3) and increased p62 (chaperone protein to autophagosome) [29] [30] [31]. BECLIN-1, a major regulator of autophagy, is decreased in IPF lung fibroblasts. Furthermore, BECLIN-1 also binds to BCL-2 and decreases apoptosis in these fibroblasts [31] [35]. As an autophagy regulator, mammalian target of rapamycin, or mTOR, kinase activity is elevated in IPF lung fibroblasts [36] via enhanced AKT activation and PTEN suppression [37]. Thus, pathways regulating autophagy in IPF likely contribute to lung fibrosis.

Previous data generated from our laboratory revealed that CSF1 contributes to pulmonary fibrosis in mice as CSF1−/−mice showed less fibrosis in response to bleomycin challenge [38]. However, the specific mechanism(s) of M-CSF-R-mediated signaling and regulatory pathways connecting CSF1 and M-CSF-R signaling to specific target cell populations is unclear. Current knowledge in the field is that AKT activation, M2 macrophage polarity, increased myofibroblast and fibrocytes numbers, and reduced autophagy are observed in the lungs of patients with IPF. Here, we extend our previous study by using a murine model, *c-fms*-directed Myr-Akt expression, to examine the role of constitutive AKT activation driven by the M-CSF-R promoter and identify potential priming of the lungs to fibrosis. With this murine model, we study a potential causal relationship between M-CSF-R-directed AKT activation and cellular phenotype to drive fibrosis. We observed that lungs from Myr-Akt mice had increased expression of genes representative of M2 macrophages, increased *α*-SMA positive cells, increased number of fibrocytes and reduced autophagy-related transcripts compared to WT mice. These data suggest M-CSF-R/AKT activation may predispose a pulmonary fibrotic phenotype.

## 2. Materials and Methods

### 2.1. Ethics Statement

This study was carried out in strict accordance with the recommendations in the Guide for the Care and Use of Laboratory Animals of the National Institutes of Health. The animal protocols were approved by the Institutional Laboratory Animal Care and Use Committee (ILACUC) of The Ohio State University (protocols: 2011A00000077 and 2009A0124-R1).

### 2.2. Reagents

Endotoxin-free RPMI 1640 (15–040) and DPBS (21–031) were purchased from Corning Cellgro. Endotoxin-free (certified less than 0.06 EU/ml) fetal bovine serum (FBS, S11150) was obtained from Atlanta Biologicals. Recombinant mouse CSF1 (416-ML) was purchased from R&D Systems. Anti-mouse CD45 antibody (clone 30-F11, 103112), Alexa488 anti-mouse F4/80 (clone BM8, 123120), PerCP/Cy5.5 anti-mouse CXCR4 (clone L276F12, 146510), and APC anti-mouse CD115 (CSF-1R/M-CSF-R) (clone AFS98, 135510) were purchased from Biolegend. Anti-rabbit *α*SMA (ab5694) and anti-mouse Collagen I (clone COL-1, ab6308) were obtained from Abcam. LC3B (D11) XP Rabbit mAb (3868) and Alexa Fluor® 488 conjugate secondary antibody was purchased from Cell Signaling Technology. FITC anti-mouse *α*-SMA antibody (clone 1A4, F3777) was purchased from Sigma-Aldrich. Cytofix/Cytoperm solution (554714) was purchased from BD Biosciences. Primers and SYBR Green Master Mix (4385610) for quantitative real-time polymerase chain reaction (qRT-PCR) were purchased from Thermofisher Scientific unless indicated otherwise. Bleomycin was obtained from Sicor Pharmaceuticals (Irvine, CA).

### 2.3. Animal Model

Constitutively-active myristoylated Myr-Akt transgenic mice were generated by Dr. Michael Ostrowski (OSU, Columbus, OH) as described previously [39]. Briefly, a *c-fms*-Myr-Akt plasmid was used to generate the mouse expressing a membrane-bound form of Akt1 in mononuclear phagocytes. The myristoylation tag on Akt facilitates membrane binding resulting in constitutive activation (Supplemental Figure 1(a)). 6 – 8 week old male and female mice were sacrificed by cardiac puncture after isoflurane inhalation and lungs perfused with 25 – 35 mL DPBS. Tissues were fixed in 10% formalin for histologic and immunohistochemical analysis, snap-frozen in liquid nitrogen for RNA analysis, or digested immediately to obtain a single cell suspension for flow cytometry analysis. Bone marrow-derived macrophages (BMM) were obtained from Myr-Akt mice or WT littermate mice by culture in RPMI 1640 media supplemented with 10% FBS, 1% PSA (Penicillin-Streptomycin Amphotericin), 5 μg/mL polymyxin B, and 20 ng/ml recombinant murine CSF1 as described [40]. 3 – 5 mice per group were used in the experiments as indicated in the Figures and Legends. Bleomycin treatment was administered as described [38] using 6 – 12 week old male mice.

### 2.4. Immunohistochemistry (IHC)

Immunohistochemical processing and staining with hematoxylin and eosin (H&E), Masson’s Trichrome, F4/80 or *α*-SMA staining were done at the Histology Core at OSU Veterinarian School as described [41]. For LC3B staining, mouse lungs were fixed in freshly made 4% paraformaldehyde for 24 hours and processed and stained at OSU Wexner Medical Center Pathology Core. Ten pictures per section were captured using the Olympus IX50 inverted microscope-equipped Nikon camera (Olympus). The average number of pixels corresponding to specific stains was quantified using Adobe Photoshop CS and histogram analysis (Adobe) as described [41]. Data are presented as percent positive cells per high-power field (HPF) and are expressed as the mean ± SEM.

### 2.5. Single Cell Suspension and Flow Cytometry

Mouse tissue were harvested and immediately homogenized with the McIlwain Tissue Chopper (Ted Pella, Inc.) to obtain a single-cell suspension as described previously [41]. Briefly, mechanically homogenized tissue was digested in RPMI 1640 medium containing 0.525 mg/mL collagenase Type 1 (Worthington Biochemical Corporation), 0.02 mg/mL Deoxyribonuclease I (Sigma), and 10% FBS in a 37°C shaker for 1 hour at 250 rpm. The homogenates were passed through a 70 μm filter and red blood cells were lysed with RBC Lysis Buffer (0.034 M aluminum chloride, 0.01 M potassium bicarbonate, 0.1 mM EDTA in dH_2_O) for 30 sec. Cells were suspended in flow wash buffer (1% BSA and 0.1% sodium azide in DPBS) and an equal number of cells subjected to flow cytometry. Cells were first incubated with Chrom Pure Mouse IgG (Jackson Immuno Research) to block Fcγ receptors. Cells were then stained with extracellular antigen antibody on ice for 30 minutes prior to permeabilization/fixation on ice for 20 minutes and intracellular staining on ice for an additional 30 minutes. Cells were washed and measured with the BD LSR II flow cytometer (BD Biosciences). Data was analyzed using FCS3 Express software (De Novo).

### 2.6. Fibrocytes Isolation

Primary murine fibrocytes were isolated from freshly collected peripheral blood as described [42]. Briefly, blood was collected from cardiac puncture and PBMCs were isolated using Lympholyte-Mammal (Cedarlene). PBMCs were cultured in FibroLife basal media (Lifeline Cell Technology) supplemented with 20 mM HEPES, 2× NEAA, 2 mM sodium pyruvate, 4 mM glutamine, 100 U/mL penicillin, 100 μg/ml streptomycin, and 2× ITS-3 for 5 days to generate mouse fibrocytes. Cells were counted and an equal number of cells were stained with antibodies specific for CD45 and CXCR4, permeabilized/fixed, and stained for Collagen I. The cells were analyzed by flow cytometry.

### 2.7. Analysis of Lung Inflammation by Pathological Assessment

A board-certified veterinary pathologist subjectively analyzed in a blinded manner and assessed the lungs for inflammation, the number of macrophages, and the amount of fibrous connective tissue present within the H&E- and Trichrome-stained slides, and scored them accordingly as previously described [38].

### 2.8. RNA Extraction and Quantitative Real-Time PCR

Total RNA was extracted using TRIzol (Thermofisher Scientific) and 1 μg total RNA was used to synthesize cDNA with SuperScript III Reverse Transcriptase (Thermofisher Scientific). Quantitative real-time (qRT)-PCR was performed in duplicate using SYBR Green Master Mix (Applied BioSystems) and an ABI PRIZM 7700 with Sequence Detector v1.7 (Applied Biosystems). Primer sequences for mouse *collagen* 1*A*, *collagen* 3*B*, *IL-1*2, *iNos*, *IL*10, *Ym*1, *Atg*3, *Atg*5, *Beclin*-1, *Lc*3*a* and *Lc*3*b* are listed in Supplemental Table 1. Target genes were normalized to the average values of housekeeping genes *Cap*1, *Rpl*4 (Thermofisher Scientific) and *Gapdh* [38] and expressed as relative copy number (RCN) using 2^(−ΔCt)^.

### 2.9. Western Blot

Single cell suspensions were obtained and lysed in Cell Lysis Buffer with protease inhibitors (Cell Signaling Technology). Protein concentration was determined by the Dc protein assay (Bio-Rad) and equal amounts of protein were separated on 10% or 4% – 12% NuPAGE Novex bis-tris gel (Thermofisher Scientific), transferred to a nitrocellulose membrane, probed with the indicated antibodies and detected by ECL (GE Healthcare Bio-Sciences Corp.). ECL signal and band density were quantified using Quantity One program (Bio-Rad). Protein expression was normalized to total protein or *β*-ACTIN.

### 2.10. Statistical Analyses

Data were analyzed using analysis of variance (one-way ANOVA) and multiplicities were adjusted by Tukey’s method to control the family-wise error rate at 0.05 using JMP 9 software (SAS Institute, Inc; Cary, NC). *P* ≤ 0.05 was defined as statistically significant in these studies. Hypothesis testing of synergistic effect were tested by interaction contrasts. Differences in survival between groups were compared by log-rank test. SAS 9.3 software (SAS Institute, Inc; Cary, NC) were used for data analysis.

## 3. Results

### 3.1. M-CSF-R Driven Myr-Akt Induces an M2 Macrophage Tropism in Lung

**Figure 1.**
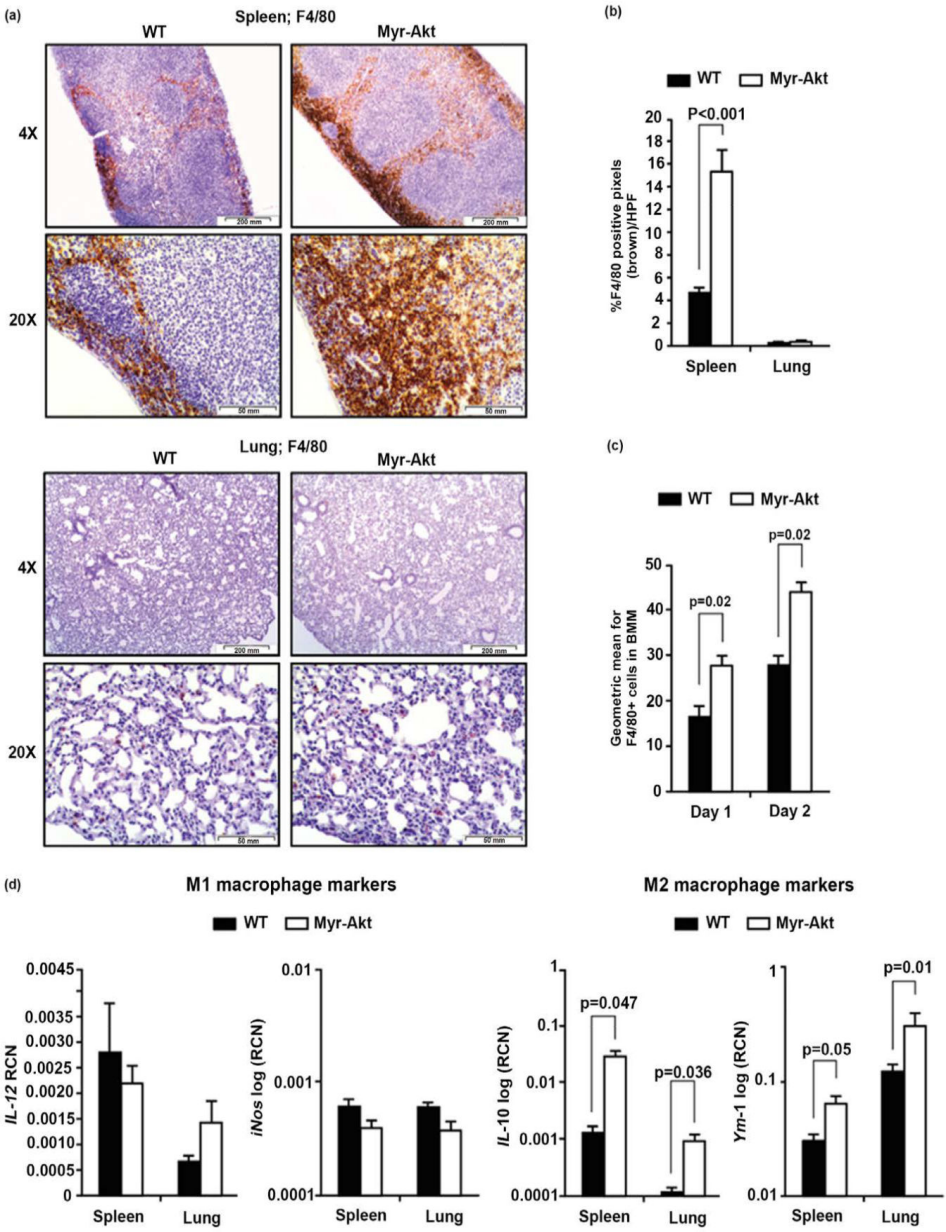
Myr-Akt mice demonstrate increased F4/80+ macrophages in the spleens and a M2 macrophage bias in the lungs. 6 – 8 week old Myr-Akt or WT mice were sacrificed and (a, *top*) spleens and (a, *bottom*) lungs were harvested, paraffin embedded, and subjected to F4/80 immunohistochemical staining for macrophages. Ten pictures per section were imaged using 4X and 20X objectives (40× and 200× magnification, respectively). (b) Images were quantified using histogram analysis feature in Adobe Photoshop CS5 software for percent brown stain (F4/80+ pixels) per high-power field (HPF) and expressed as mean ± SEM. (c) BMMs were generated from Myr-Akt or WT littermate mice with recombinant murine (rh)CSF1 (20 ng/ml). Cells were removed using Accutase one and two days after seeding the progenitor cells (day 0). Cell surface antigen expression of F4/80 was assessed by flow cytometry. Data presented are the average geometric mean for F4/80 expression ± SEM. (d) Total RNA was extracted from lung and spleen using TRIzol and cDNA synthesized from 1 μg RNA for quantitative real-time (qRT)-PCR using primers specific for mouse *IL*12, *iNos*, *IL*10, or *Ym*1. Data are expressed as relative copy number (RCN) (d, *left*) *IL*12 and *iNos* mRNA, or (d, *right*) *IL*10 mRNA and *Ym*1 mRNA expression over the mean of endogenous control RNAs (*Cap1*, *Rpl*4 and *Gapdh)*. Data represents the mean RCN ± SEM. 5 pairs of age-matched littermate mice were used for each experiment.

Since bone marrow-derived macrophages (BMM) collected from mice expressing Myr-Akt driven by the M-CSF-R promoter had increased survival [40], we first checked if Myr-Akt mice produced more BMM than WT mice. Penetrance of the Myr-Akt transgene correlated with transgene expression and splenomegaly. For this reason, classification as Myr-Akt genotype in this study was confirmed using both murine genotyping (Supplemental Figure 1(a)) and spleen size (Supplemental Figure 1(b)). Immunohistochemical staining for the macrophage marker F4/80 revealed a significant increase in macrophage numbers in the spleens of Myr-Akt mice compared to WT littermates (p < 0.001) (Figure 1(a), *top* and Figure 1(b)), however, we did not observe any differences in F4/80 expression in the lungs between these Myr-Akt and WT mice (Figure 1(a), bottom and Figure 1(b)). We next derived macrophages from bone marrow and measured F4/80 expression with flow cytometry. Our data suggest that Myr-Akt mice have increased numbers of F4/80+ BMMs compared to WT mice at day 1 (p = 0.02) and day 2 (p = 0.02) (Figure 1(c)). However, BMM numbers were similar after day 3 in culture (*data not shown*).

Since we did not observe more macrophages in the lungs of the Myr-Akt mice, we next asked if tissue macrophages from the lungs or spleens from Myr-Akt mice expressed a phenotype representing M2 phenotype [43] [44]. We isolated total RNA from whole lung or spleen tissue and performed qRT-PCR for the expression of M1 and M2 genes. There was no significant difference between Myr-Akt and WT mice in the M1 pro-inflammatory markers *IL*-12 and *iNos* (Figure 1(d), *left*), but there were significant increases in M2 anti-inflammatory genes *IL*-10 (*p* = 0.047 for lung and p = 0.036 for spleen) and *Ym1* (*p* = 0.01 for lung and *p* = 0.05 for spleen) of Myr-Akt compared to WT mice (Figure 1(d), *right*). Our data suggests that AKT activation driven by the M-CSF-R promoter in Myr-Akt mice drives an M2 tropism in both lung and splenic macrophages.

### 3.2. *α*-SMA+ and *α*-SMA+/M-CSF-R+ Cell Influx in the Lungs of Unchallenged Myr-Akt Mice

**Figure 2.**
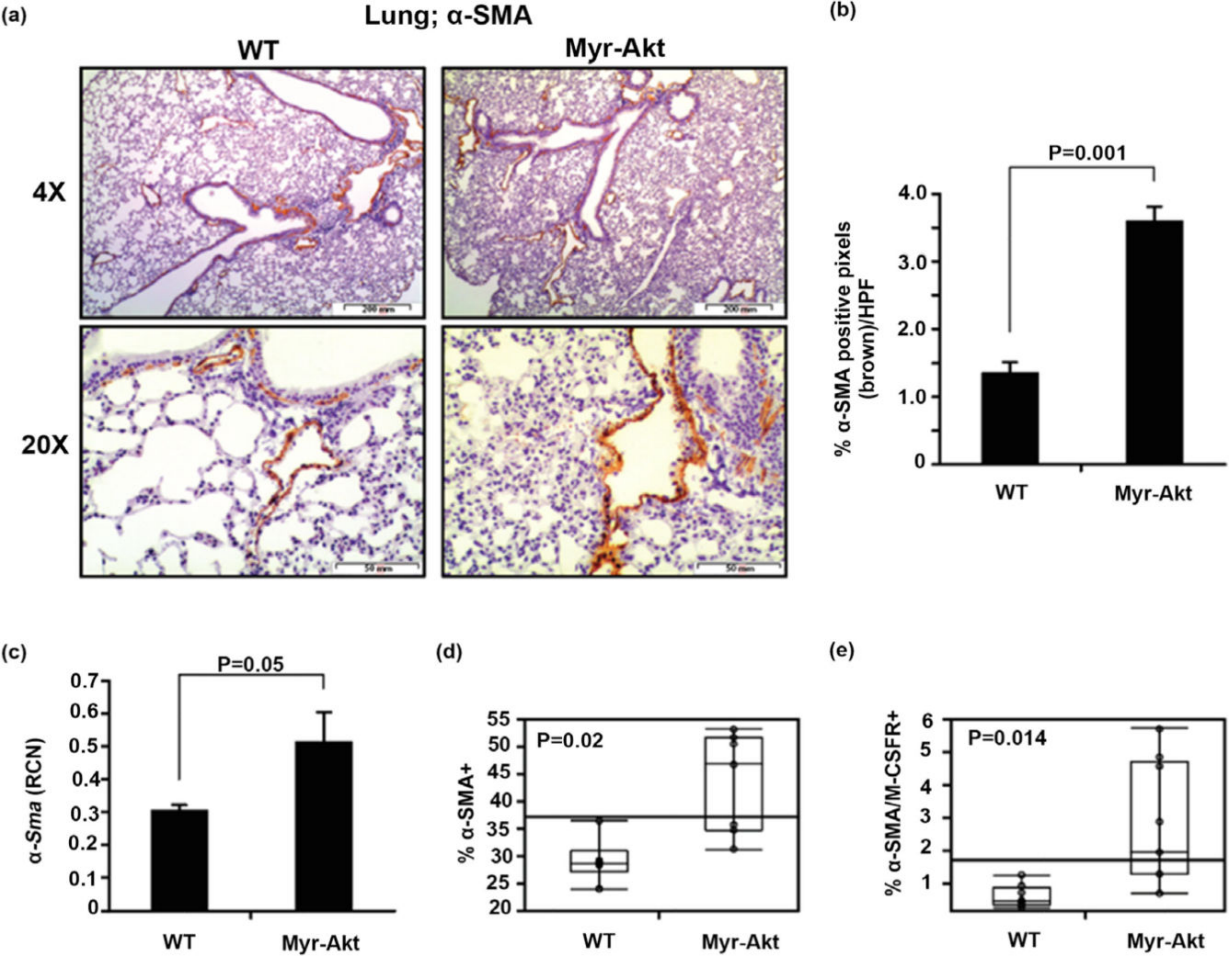
Differential myofibroblast expression in the lungs of Myr-Akt and WT mice. (a) 6 – 8 week old Myr-Akt or WT mice were sacrificed and lungs harvested, paraffin embedded, and subjected to *α*-SMA immunohistochemical staining for myofibroblasts. Ten pictures per section were imaged using 4X and 20X objectives (40× and 200× magnification, respectively). (b) Images were quantified by histogram analysis using Adobe Photoshop CS5 software and percent brown stain (*α*-SMA+ pixels) per high-power field (HPF) from at least five images per section are expressed as mean ± SEM. (c) Total RNA was extracted from Myr-Akt or WT littermate mouse lungs and *α*-SMA mRNA was measured by qRT-PCR. Data are expressed as relative copy number (RCN) of *α*-SMA mRNA expression over the average of endogenous control RNAs (*Cap*1, *Rpl*4 and *Gapdh*). Data represent the mean RCN ± SEM. Single-cell suspension from lung homogenate were immunostained using mouse α-SMA antibodies or double-immunostained with *α*-SMA and M-CSF-R (CD115) antibodies and analyzed by flow cytometry. (d) Data represents mean percent of *α*-SMA+ cells and (e) *α*-SMA+/M-CSF-R+ cells ± SEM. 5 pairs of age-matched littermate mice were used for each experiment.

Since *α*-SMA positive myofibroblasts mediate pulmonary fibrosis in human IPF [21] [45]. We next wanted to determine basal differences in *α*-SMA expression in lung cells [46] from Myr-Akt and WT mice. Using immunostaining with the myofibroblast marker *α*-SMA, we found more *α*-SMA expression in lung tissue of Myr-Akt mice than WT mice (*p* = 0.001) that were mostly confined in the epithelial and subepithelial tissues (Figure 2(a) and Figure 2(b)). This observation is supported by a significant increase in *α-Sma* mRNA expression from whole lung tissue of Myr-Akt compared to WT mice (*p* = 0.05) (Figure 2(c)). Using flow cytometry, we also found increased numbers of cells expressing *α*-SMA in the lungs of Myr-Akt mice compared to WT mice (mean 43.44% ± 3.46% for Myr-Akt vs. 29.34% ± 1.64% for WT; *p* = 0.02) (Figure 2(d)). Since the Myr-Akt isoform is controlled by the M-CSF-R promoter, we next examined the population of *α*-SMA+ myofibroblasts co-expressing M-CSF-R. We observed a significant increase in α-SMA+/M-CSF-R+ cells in the lungs of Myr-Akt mice compared to WT mice (mean 2.76% ± 0.54% for Myr-Akt vs. 0.9% ± 0.11% for WT; p = 0.014 (Figure 2(e)). These data suggest the Myr-Akt mice had increased basal concentrations of pulmonary myofibroblasts (*α*-SMA+) co-expressing M-CSF-R than WT mice.

### 3.3. Myr-Akt Mice have Altered Expression of Key Autophagy Genes in the Lung

**Figure 3.**
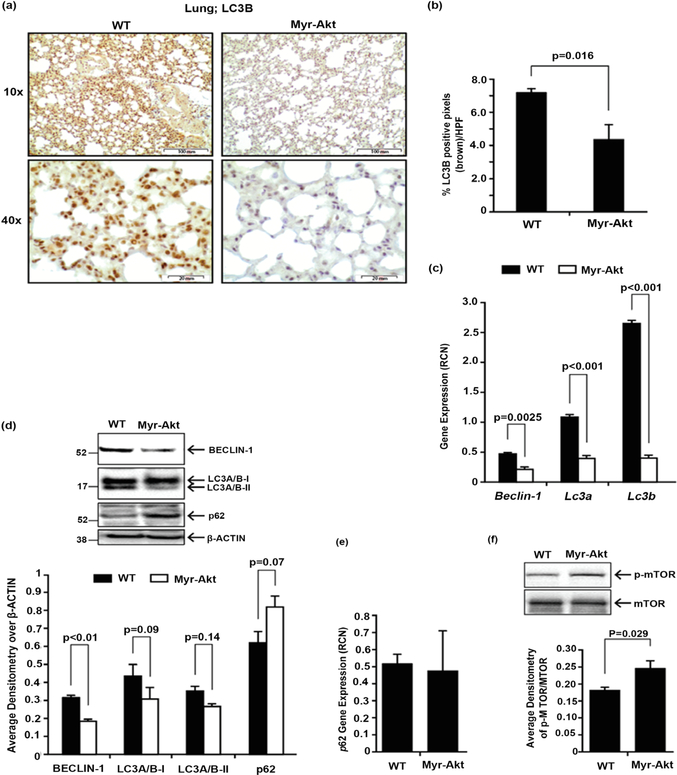
Reduced autophagy in the lungs of Myr-Akt mice. (a) 6 – 8 week old Myr-Akt or WT mice were sacrificed and lungs harvested, paraffin embedded, and subjected to LC3B immunohistochemical staining. Ten pictures per section were imaged using 10X and 40X objectives (100× and 400× magnification, respectively). (b) Images were quantified using histogram analysis feature in Adobe Photoshop CS5 software for percent brown stain (F4/80+ pixels) per high-power field (HPF) from at least five images per section and expressed as mean ± SEM. (c) Total RNA was extracted from homogenized lungs using TRIzol. cDNA was synthesized and qRT-PCR performed using primers specific for mouse *Beclin-1*, *Lc*3*a*, and *Lc*3*b* mRNAs. Data are expressed as relative copy number (RCN) of mRNA expression over average endogenous control RNAs (*Cap*1, *Rpl*4 and *Gapdh*). Data represents the mean RCN ± SEM. (d) Total protein were isolated and equal amounts of protein separated on a 4% – 12% SDS-PAGE gel and immunoblotted for BECLIN-1, LC3A/B-I and LC3A/B-II, and *β*-ACTIN. (e) Total RNA was extracted from homogenized lungs using TRIzol. cDNA was synthesized and qRT-PCR performed using primers specific for mouse *p*62/*SQSTM1* mRNA. Data are expressed as relative copy number (RCN) of mRNA expression over average endogenous control RNAs (*Cap*1, *Rpl*4 and *Gapdh*). Data represents the mean RCN ± SEM. (f) Immunoblot for phosphor-mTOR (ser2448) and mTOR (Cell Signaling Technology). Bands were quantified using densitometry by comparing band density over *β*-ACTIN or total mTOR in each respective lane. Data are expressed as the mean densitometry value ± SEM. 4 pairs of age-matched littermate mice were used for western analysis and 5 pairs of age-matched littermate mice were used for gene expression.

Autophagy is a process that maintains cell and tissue homeostasis by degrading and recycling cellular proteins and organelles during times of cellular stress. Autophagy activity is associated with organismal longevity and is reduced in lung tissue of IPF patients [29] [30] [31]. Because cellular autophagy is inhibited by the activation of the PI3K/AKT/mTOR pathway [47] [48] [49], we asked whether M-CSF-R-driven Myr-Akt impaired cellular autophagy in the lungs of the Myr-Akt mice. Following synthesis, the carboxy-terminal region of microtubule-associated protein light chain proLC3 is cleaved by the cysteine protease ATG4 generating the soluble cytosolic form of LC3A/B-I during autophagy, LC3A/B-I is converted to a membrane-bound LC3A/B-II form [50]. Since Cell Signaling Technology LC3B antibody mainly recognizes LC3BII, the hallmark of autophagy, we stained lung of Myr-Akt mice and their WT littermates with LC3B (Figure 3(a) and Figure 3(b)). Interestingly, we observed a significant decrease in LC3B in lungs from Myr-Akt mice compared to lungs from WT mice (p = 0.016), implying autophagy is impaired in the lung of Myr-Akt mice. We next analyzed key autophagy-regulating genes: *Beclin*-1 [29] and *Lc*3*a* and *Lc*3*b* [50] [51] by qRT-PCR. *Beclin*-1, *Lc*3*a* and *Lc*3*b* gene expression were significantly reduced in lungs from Myr-Akt mice compared to lungs from WT mice (p = 0.0025 for *Beclin*-1, and p < 0.001 for *Lc*3*a* and *Lc*3*b*) (Figure 3(c)). Similarly, BECLIN-1 and LC3A/B-II protein was also significantly reduced in the lungs of Myr-Akt mice compared to lungs from WT mice (p < 0.01 for BECLIN-1, p = 0.09 for LC3A/B-I and p = 0.014 for LC3A/B-II) (Figure 3(d)). Because inhibition of autophagy increases autophagy receptor p62 protein accumulation [52], we also assessed the expression of polyubiquitin-binding protein p62/SQSTM1 and observed an increased accumulation of p62 protein in the lungs of Myr-Akt mice (Figure 3(d), p = 0.07). Even though p62 protein serves as an autophagy marker, it can be regulated at both transcriptional and post-translational levels [53], therefore we measured p62 mRNA by qRT-PCR from Myr-Akt and WT mice lungs and found no change on p62 mRNA, indicating that p62 is regulated at a post-translational level and the accumulation of p62 protein is due to low autophagy (Figure 3(e)). Further, since AKT positively regulates mTOR activity, we confirmed an elevation of phosphor-mTOR in the lungs of Myr-Akt mice (Figure 3(f), p = 0.029). Taken together, these data suggest that the lungs of Myr-Akt mice have reduced basal autophagy and increased mTOR activity compared to lungs of WT mice.

### 3.4. Myr-Akt Mice Produce Increased Numbers of Circulating Fibrocytes *in Vivo*

**Figure 4.**
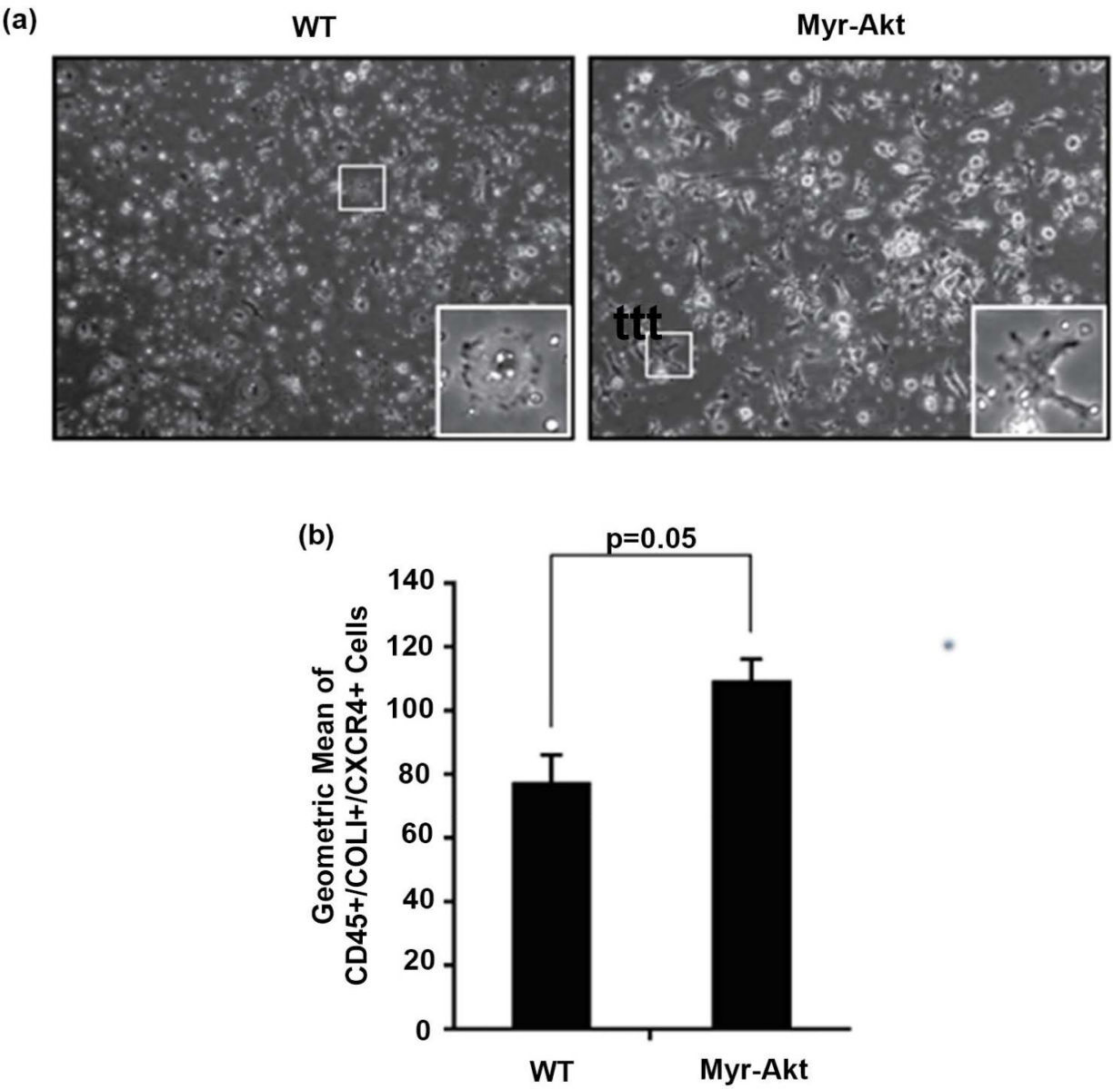
Myr-Akt mice have elevated circulating fibrocytes compared to WT mice. (a) 6 – 8 week old Myr-Akt or WT mice were sacrificed and blood was collected from cardiac puncture. PBMCs were isolated and cultured in FibroLife media for 5 days to generate mouse fibrocytes. Images were taken at day 5 using a 10X objective (100× magnification). (b) Cells were removed using Accutase and equal number of cells were stained with CD45 and CXCR4, then permeabilized/fixed and stained for Collagen I. The cells were analyzed by flow cytometry and fibrocytes were identified as CD45+/COLI+/CXCR4+ populations. Data represents mean percent of fibrocytes ± SEM from 5 mice per group.

Bone-marrow derived circulating fibrocytes can traffic to the lung during the pathogenesis of pulmonary fibrosis [24] [28]. We inquired whether Myr-Akt mice could generate more circulating fibrocytes, *in vivo*. We generated fibrocytes from mouse PBMCs cultured in fibrocyte-deriving medium and observed more spindle-shaped fibroblast-like cell morphology from Myr-Akt mice compared with WT mice (Figure 4(a)). Since CD45+/COL1+/CXCR4+ fibrocytes were expanded in the bone marrow and have been reported to contribute to bleomycin-induced pulmonary fibrosis [24], we next stained the PBMC-differentiated cells with antibodies specific for CD45, Collagen I, and CXCR4 (Figure 4(b)). Our data suggest Myr-Akt mice generate significantly more circulating CD45+/COL1+/CXCR4+ fibrocytes than WT mice (p = 0.05), basally.

### 3.5. Bleomycin Challenge Enhances Collagen Deposition in the Lungs and Reduces Survival in Myr-Akt Mice

Because Myr-Akt macrophages express an M2 tropism, increased *α*-SMA+ myofibroblasts, increased circulating CD45+/COL1+/CXCR4+ fibrocytes, and reduced autophagy-related gene expression, we hypothesized that Myr-Akt mice would develop more fibrosis in response to repeated administration of bleomycin.

**Figure 5.**
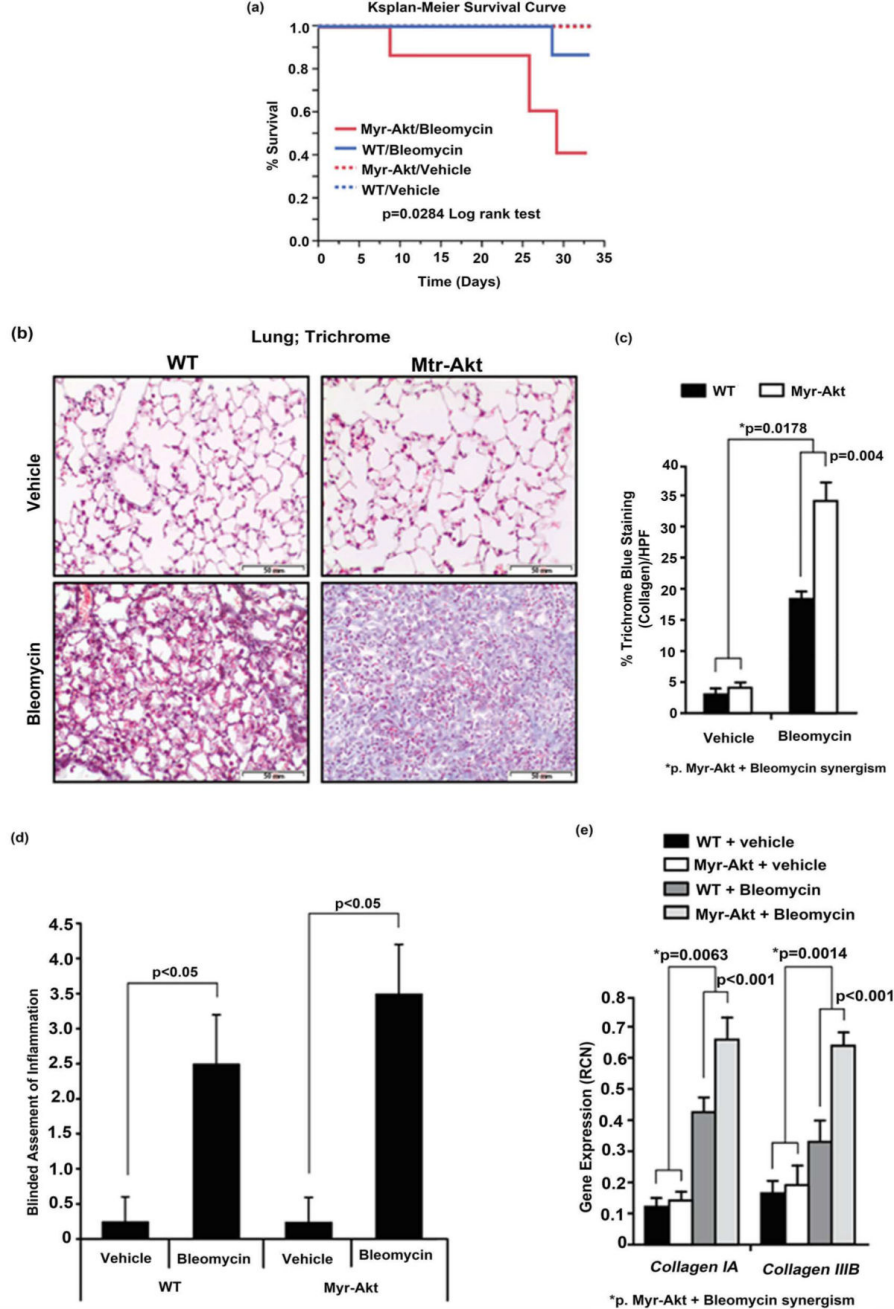
Bleomycin induces collagen deposition in the lungs and reduces survival in Myr-Akt mice. Myr-Akt and wild type (WT) littermate mice were treated twice a week with bleomycin (0.035 U/g) or PBS (vehicle) for 4 weeks, as described in Material and Methods. (a) Mouse survival was recorded and plotted using a Kaplan-Meier curve. Myr-Akt mice had worse survival than WT mice in response to bleomycin treatment. N = 7 mice for each WT and Myr-Akt mouse group. (b) All surviving mice were sacrificed at day 33 and the lungs formalin-fixed, sectioned, and stained with Masson’s Trichrome. Five images per slide were captured. Red staining represents keratin, light red and pink staining represents cytoplasm, dark brown represents cell nuclei, and blue staining represents collagen. The images are representative images from 3 mice per group using a 20X objective (200× magnification). (c) The images were quantified blindly by histogram analysis using Adobe Photoshop CS and the percent of blue staining per high-power field (HPF) from ten images and expressed as mean ± SEM. (d) Lungs from WT and Myr-Akt mice subjected to PBS or bleomycin stained with H&E, Trichrome, and F4/80 were evaluated by a board-certified pathologist for inflammation score (0 to 5 point scale). (e) Total RNA was extracted from homogenized lung tissue using TRIzol. cDNA was synthesized and qRT-PCR performed for mouse collagen IA and IIIB. Data are expressed as mean relative copy number (RCN) of mRNA expression over average endogenous control RNAs (*Cap*1, *Rpl*4 and *Gapdh*). Data represents the mean RCN ± SEM and N = 4 mice per group.

Kaplan-Meier survival analysis (Figure 5(a)) illustrates decreased survival in Myr-Akt mice challenged with bleomycin compared to bleomycin-treated WT mice (*p* = 0.0284). While there was no mortality in the Myr-Akt or WT mice groups treated with PBS (vehicle), the Myr-Akt mice started to die at day 7 post-bleomycin treatment while all WT mice survived until day 28 on the bleomycin protocol. The mice receiving bleomycin and surviving to day 33 showed typical fibrosis in the lung compared to vehicle-treated mice, including blue stained pixels (Trichrome) as an indication of collagen deposition (Figure 5(b) and Figure 5(c)) and significant inflammation (Figure 5(d)). Bleomycin increased collagen deposition in the lungs of WT mice by 15% (bleomycin vs. vehicle in WT, p < 0.001), while Myr-Akt increased the bleomycin effect to 28% (bleomycin vs. vehicle in Myr-Akt, p = 0.004), and a statistical synergistic test showed that this enhancement was significant (p = 0.0178). Moreover, bleomycin treatment up-regulated collagen IA and IIIB mRNAs by 4-fold and 3-fold in WT (both *p* < 0.001, compared with vehicle), while Myr-Akt increased the bleomycin effect to 6-fold and 5-fold, respectively (bleomycin vs. vehicle in Myr-Akt, both p < 0.0001). A statistical synergistic test showed that this enhancement was significant (p = 0.0063 for collagen IA, p = 0.0014 for collagen IIIB) (Figure 5(e)). Our results suggest that Myr-Akt enhances the bleomycin effect leading to the increased lung fibrosis and decreased overall survival.

### 3.6. Myr-Akt mice Demonstrate Increased CD45 and F4/80+ Cells after Bleomycin Challenge

**Figure 6.**
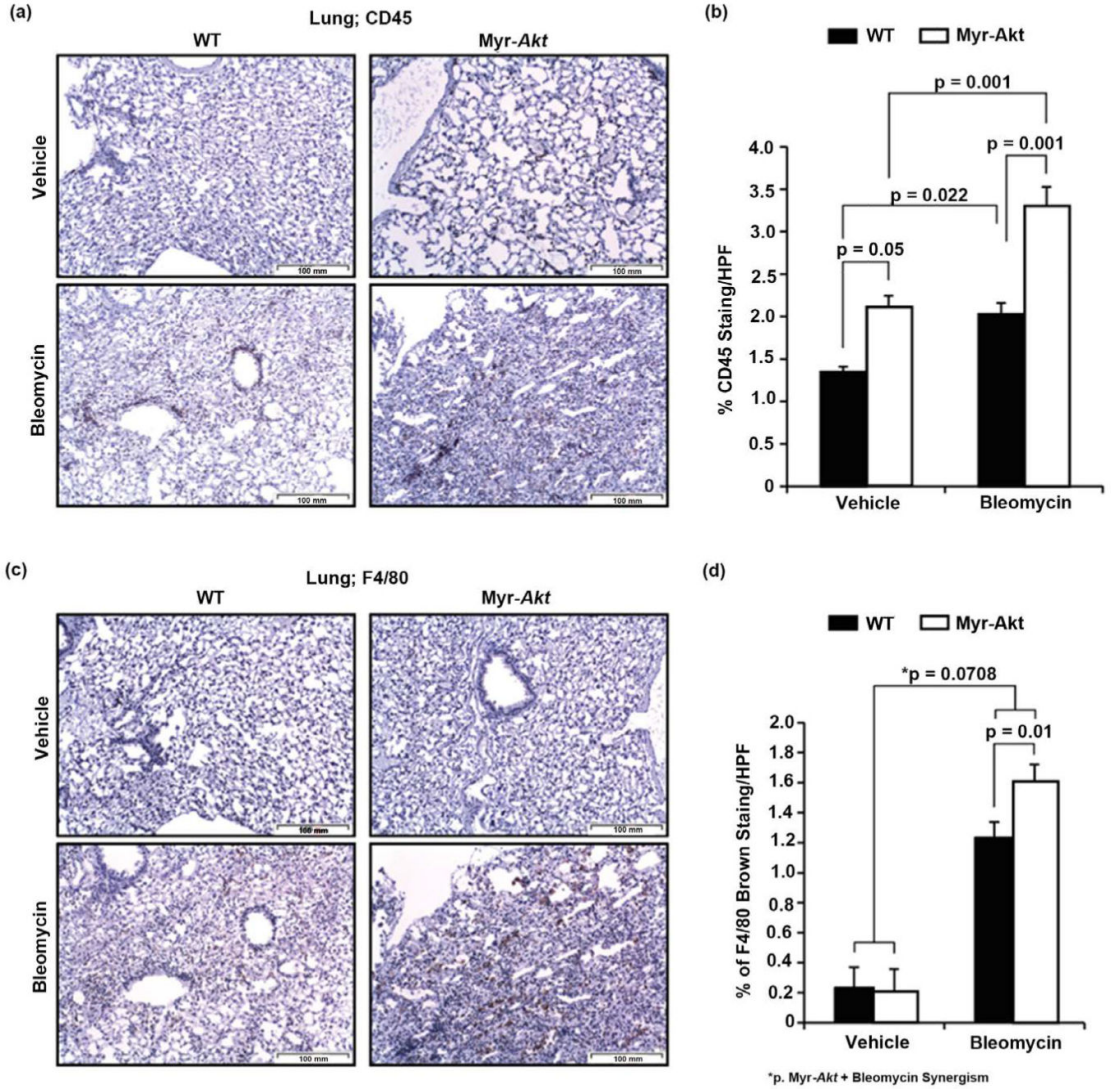
Bleomycin increases CD45+ and F4/80+ cells in the lungs of Myr-Akt mice. Mice surviving the bleomycin or PBS protocol were sacrificed at 33 days and lungs harvested and subjected to immunohistochemical staining. Ten pictures per slide were imaged. Brown pixels represent (a) CD45+ or (c) F4/80+ cells. Images are representative of three mice per group. The images were quantified blindly by histogram analysis using Adobe Photoshop CS and the percent brown stain per high-power field (HPF) from at least five images per section using a 10X objective (100× magnification) and are expressed as the mean ± SEM (b) and (d).

To determine if Myr-Akt mice had enhanced lung inflammation, we immunostained mouse lungs using the leukocyte marker CD45 [54] and tissue macrophage marker F4/80 [16]. More CD45+ cells are present in Myr-Akt mice compared with WT (p = 0.05) (Figure 6(a) and Figure 6(b)). As expected, bleomycin treatment significantly increased CD45+ cells in the lungs of WT and Myr-Akt mice (both p < 0.01). Additionally, more CD45+ cells were present in the lungs of Myr-Akt mice compared to the lungs of WT mice after bleomycin challenge (*p* = 0.001) (Figure 6(a) and Figure 6(b)). Importantly, unlike CD45+ cells, and as seen in Figure 1(a) and Figure 1(b), the F4/80+ cells are comparable between Myr-Akt and WT mice (Figure 6(c) and Figure 6(d)). After bleomycin challenge, more F4/80+ cells were present in the lungs of Myr-Akt mice than WT mice (p = 0.01). As expected, bleomycin increased F4/80+ cells in the lungs of WT mice by 6-fold (*p* < 0.001), while Myr-Akt increased the bleomycin effect by 8-fold (bleomycin vs. vehicle in Myr-Akt). Although not quite statistically different, Myr-Akt and bleomycin exerted a marked synergism on F4/80 expression (*p* = 0.0708) (Figure 6(d)). These data suggest that bleomycin-induced lung fibrosis may involve recruited hematopoietic cells, specifically M2 macrophages.

### 3.7. Bleomycin Suppresses Autophagy Gene Expression in the Lungs

**Figure 7.**
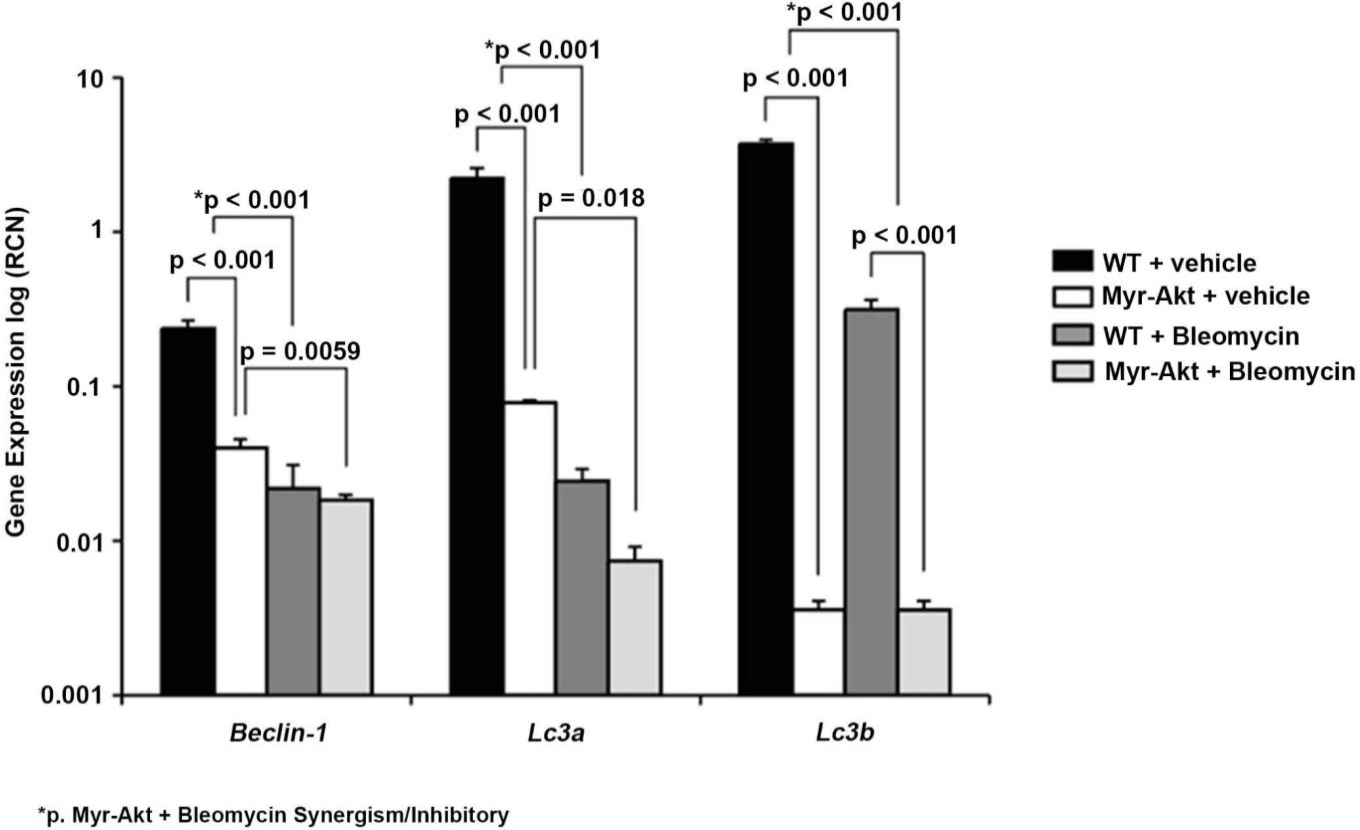
Bleomycin suppresses autophagy gene expression in the lungs of Myr-Akt mice. Total RNA was extracted from paraffin embedded lung tissue section with RecoverAll Total Nucleic Acid Isolation Kit for FFPE according to manufacturer’s protocol. cDNA was synthesized and qRT-PCR performed for mouse *Beclin-*1, *Lc*3*a*, and *Lc*3*b*. Data are expressed as relative copy number (RCN) of mRNA expression over average of endogenous control RNAs (*Cap*1, *Rpl*4 and *Gapdh*.). Data represents the mean RCN ± SEM and N = 4 mice per group.

Reduced levels of autophagy in IPF induce epithelial cell senescence and accelerate lung fibroblast differentiation into myofibroblasts [29]. Our data suggest that Myr-Akt mice both basally and post-bleomycin contained more *α*-SMA+ lung myofibroblasts and fewer autophagy-related genes expressed in lung tissue than WT mice. Specifically, we initially observed (Figure 3) that basal Myr-Akt expression decreased the key autophagy genes *Beclin*-1, *LC*3*a* and *LC*3*b* compared to WT. This data was confirmed as vehicle-treated Myr-Akt mice had significantly reduced *Beclin*-1, *LC*3*a* and *LC*3*b* mRNAs in whole lung tissue compared to WT mice (*p* < 0.001 for *Beclin*-1, *LC*3*a*, and *LC*3*b*). Predictably, bleomycin treatment in both WT and Myr-Akt mice further reduced *Beclin*-1 mRNA (*p* < 0.001 for vehicle vs. bleomycin in WT mice; *p* < 0.006 for vehicle vs. bleomycin in Myr-Akt mice); *LC3a* mRNA (*p* < 0.001 for vehicle vs. bleomycin in WT mice; *p* < 0.001 for vehicle vs. bleomycin in Myr-Akt mice); and *LC3b* mRNA (*p* < 0.001 for vehicle vs. bleomycin in WT mice; *p* < 0.001 for vehicle vs. bleomycin in Myr-Akt mice) compared to sample-matched vehicle control (Figure 7). Interestingly, a synergistic decrease of autophagy genes was observed after bleomycin treatment in Myr-Akt mice (p < 0.001 for beclin-1, LC3a and LC3b) (Figure 7).

## 4. Discussion

We previously reported that CSF1 plays an important role in pulmonary fibrosis in both mice and IPF patients [38]. However, that report lacked critical mechanistic data about how CSF1 and M-CSF-R-mediated signaling contributes to lung fibrosis. To accomplish an understanding of how M-CSF-R and AKT activation may contribute to fibrosis, we used a novel transgenic mouse model which expresses a myristoylated and constitutively-active AKT isoform under the control of the M-CSF-R promoter to investigate the connections between cellular CSF1 signaling and the development of pulmonary fibrosis. Interestingly, we did not observe basal structural differences in the lungs of the transgenic or wild type mice as shown by Hematoxylin & Eosin (H&E) staining (Supplemental Figure 2(a)). However, compared to lung tissue from WT mice, Myr-Akt mice lungs contained macrophages that expressed a basal M2 tropism, exhibited a higher number of lung cells co-expressing M-CSF-R and *α*-SMA, contained increased numbers of circulating fibrocytes, and had reduced key autophagy gene expression: *Lc*3*a/b*, *Beclin*-1 and p62 at both the mRNA and protein levels. Further, when challenged with bleomycin, Myr-Akt mice had more lung fibrosis, inflammation, myofibroblasts, less autophagy and shorter life span than WT mice given bleomycin. Interestingly, we observed a synergistic effect of Myr-Akt expression and bleomycin challenge on cellular and molecular pathways that regulate lung pathology and fibrosis. Our study provides insight into understanding a critical role for CSF1-induced AKT activation in the predisposition to lung fibrosis.

While we are excited about the potential of M-CSF-R and AKT targeting in lung fibrosis, we acknowledge limitations in the Myr-Akt mouse as a model for human lung fibrosis. First, the penetrance of Myr-Akt expression is difficult to ascertain in these mice solely by copy number. Thus, to select mice that had maximum penetrance of the Myr-Akt construct, we first genotyped the mice and then classified those Myr-Akt mice with enlarged spleens as Myr-Akt mice displaying the constitutively-active AKT phenotype as splenomegaly is a characteristic of enhanced macrophage accumulation in these mice when compared to wild type mice. We further stained spleen with the macrophage marker F4/80 and confirmed Myr-Akt mice had significantly higher level of F4/80 accumulation in the spleen than WT mice. Finally, we assayed lungs, spleens, and bone marrow from both groups of mice and found augmented phosphorylation of AKT and ERK in the Myr-Akt mice (Supplemental Figure 3). Due to technical limitations, we could not effectively obtain enough M-CSF-R+ cells from mouse lungs. We would expect to observe greater differences at the gene and protein levels between Myr-Akt mice and WT mice with M-CSF-R+ cells.

This unique model provides insight into the role of M-CSF-R in lung fibrosis. Despite the limitations of mouse models to emulate human lung fibrosis, we gained valuable insight from this study. First, M-CSF-R-induced AKT activation enhanced basal lung M2 macrophage tropism in Myr-Akt mice suggesting that activation of M-CSF-R- and AKT-driven genes may predispose a host to a fibrotic response after secondary insult or injury. In human fibrotic organ tissue, M2 macrophages contribute to the regulation of inflammation, tumor growth, stromal formation, and clearance of cell debris in the ECM [16].

In addition to our observations about the role of M-CSF-R activation in IPF [38], another study demonstrated elevated M2 genes *CCL*18, *CCL*22, and *CD*206 from alveolar macrophages of IPF patients. In this study, the authors proposed *L-*4 and *IL-*10 are responsible for the M2 phenotype shift [55]. Most importantly, our findings suggest a link between M-CSF-R-regulated AKT activation and an M2 macrophage polarity as whole lung tissue from Myr-Akt mice had increased IL-10 mRNA and an M2 macrophage polarization in both lungs and spleens. Admittedly, alveolar macrophages are not the only producers of IL-10, and increases in IL-10 mRNA from whole lung tissue could be from other inflammatory cells as well as type I alveolar epithelial cells in this model. Studies are underway to confirm the sources of lung IL-10 in the mouse model. Indeed, the negative regulator of PI3K/AKT, src homology 2-containing inositol phosphatase (SHIP), interferes with lung M2 macrophage skewing as peritoneal and alveolar macrophages from SHIP1−/− mice express a strong M2 gene signature in terms of *arginase-1* and *Ym-1* mRNAs along with M2-mediated lung pathology [44]. Bone marrow-derived macrophages from SHIP1−/− mice display an M2 phenotype only upon TGF-*β* or IL-3 stimulation [14] [43]. Furthermore, the PI3K inhibitor LY294002 decreases the expression of M2 genes from alveolar macrophages [2] [14]. It is interesting that we did not observe any significant morphological changes or increase in macrophage numbers in the lungs from the Myr-Akt compared to WT mice (Figures 1(a), *bottom* and Figures 1(b)). Therefore, our data of macrophage M2 phenotype tropism, increased myofibroblast and circulating fibrocytes population, and reduced autophagy genes suggest a potential priming effect driven by M-CSF-R and AKT activation that favors a lung environment that is primed for fibrosis.

The current paradigm in pulmonary fibrosis suggests that in a predisposed host, repeated subclinical lung injury and epithelial damage results in recruitment and activation of highly contractile and fibrotic myofibroblasts [2] [56]. Interestingly, phosphatase and tensin homolog (PTEN), a negative regulator of AKT activity, is down-regulated in myofibroblasts [56] isolated from IPF patients [9]. We observed elevated numbers of basal *α*-SMA+ myofibroblasts in untreated Myr-Akt lungs. Moreover, myofibroblasts have been shown to be more resistant to apoptosis than normal fibroblasts, which may interrupt epithelial cell repair [19] [30] [57]. As constitutively-active AKT was observed in spleens, lungs, and bone marrow of Myr-Akt mice (Supplemental Figure 3), we speculated that persistent AKT activation may lead to myofibroblast survival, differentiation, and ultimately organ fibrosis. Of note, we observed increases in *α*-SMA/M-CSF-R double-positive cells in the lung of Myr-Akt mice. This observation suggests that as part of the priming effect, either proliferating smooth muscle cells known to express M-CSF-R [22] differentiate into myofibroblasts, or macrophages possibly trans-differentiate into myofibroblasts. These questions require further investigation to clarify the hematopoietic or mesenchymal origin of these cells.

Increasing evidence indicates detection of fibrocytes in fibrotic lungs. Fibrocytes are important sources of fibroblasts and myofibroblasts during tissue repair and remodeling processes [24]. Indeed, a key marker for fibrocytes, CXCR4 expression, is regulated by the PI3K/AKT/mTOR pathway and hypoxia as inhibiting PI3K/AKT/mTOR activity reduced PDGF and hypoxia-induced CXCR4 [27]. These data support increases in circulating fibrocytes in our Myr-Akt mice. Interestingly, a recent report suggests that fibrocytes do not contribute to significant type I collagen deposition in fibrotic lungs and that they may play an indirect role in fibrosis by orchestrating recruitment of other cells [58]. Studies to investigate the role of AKT in the differentiation of macrophages into fibroblasts and their paracrine effects on fibroblasts are ongoing.

In addition to the predisposed M2 polarity, and myofibroblast and fibrocyte presence, we also found cells from lungs of Myr-Akt mice had altered expression of key autophagy-regulatory genes, indicating reduced autophagy. Since autophagy is a critical pathway for cellular longevity and homeostasis, and is reduced in lungs of IPF patients, understanding the molecular regulation of this pathway may contribute to effective pharmacological intervention. Based on existing literature, we chose to measure LC3-II (lipidated/autophagosome membrane associated form of LC3), Beclin-1 and p62 (ubiquitin binding autophagy substrate receptor) proteins as markers for autophagic. Use of three markers to assess autophagic helps overcome the limitations of any one single marker in assessing such a complex system, facilitating more accurate interpretation of results. Our results indicate that lung tissue from Myr-Akt mice had reduced autophagy. Consistent with our observation in Myr-Akt mice, others demonstrate that inhibiting autophagy by knocking down *Lc*3*b* and *Atg*5 mRNAs enhance expression of *α*-SMA and type I collagen in lung fibroblasts [29]. Similarly, inducing autophagy by inhibiting mTOR activity in lung fibroblasts with rapamycin reduces cellular *α*-SMA and fibronectin expression [30] linking autophagy and *α*-SMA expression with lung fibrosis in Myr-Akt mice. Of note, in addition to qRT-PCR for expression of lung collagen mRNAs, we elected to use Trichrome staining in lieu of Sircol or hydroxyproline assay on whole lung tissue to determine potential difference in location of collagen deposition. Further, we failed to assess autophagic flux with lysosome inhibitors, *in vivo*. Autophagic flux is the complete process of autophagy from phagophore formation to substrate degradation and release of breakdown products. Measuring autophagy flux will be included in our future autophagy analysis.

Interestingly, the regulation of autophagy may be cell-specific. Araya et al. reported that activating autophagy in human bronchial epithelial cells suppresses endoplasmic reticulum (ER) stress and cell senescence [29]. In contrast, inhibiting autophagy also promotes myofibroblast differentiation in the lungs of IPF patients [29]. Our study showed a reduction in key autophagy genes, *Beclin*-1, *Lc*3*a* and *Lc*3*b* in Myr-Akt mouse lungs. Several lines of evidence indicate that autophagy, apoptosis, and senescence are tightly regulated to maintain cellular and organ homeostasis. For example, reduced apoptosis in lung myofibroblasts from human IPF samples results in dysregulated lung repair [59] correlating with the inhibition of autophagy in IPF lung tissue [30] [31] [47]. Importantly, AKT-induced mTOR activation is critical in reducing autophagy as rapamycin has been shown to induce autophagy [47]. We observed elevated mTOR activity in lungs of Myr-Akt mice suggesting decreased autophagy via the AKT/mTOR pathway. Furthermore, in our Myr-Akt murine model, constitutive AKT activation correlated with reduced autophagy gene expression, which was further reduced by bleomycin challenge. It is plausible that the combination of constitutively-active AKT and bleomycin creates a scenario where clearance of unwanted proteins and organelles is hindered, leading to reduced survival times in Myr-Akt mice. We are currently investigating if AKT activation in macrophages, and the observed changes in lung expression of autophagy markers, are directly attributable to changes in the macrophages themselves or if this AKT activity translates into alterations of other cell phenotypes.

This report provides links between M-CSF-R-induced AKT activity and M2 macrophage bias, myofibroblast marker expression, and decreased autophagy gene expression. We used a transgenic murine model to demonstrate a pro-fibrotic role for CSF1 and AKT in the lungs, and believe that these observations may be applicable to clinical lung disease, as we showed overexpressing AKT predisposes lungs to fibrosis in the presence of bleomycin. This prediction is based on our previous data that showed CSF1 is important in IPF, and we surmise that this report clarifies a potential mechanism for this effect as a priming induced by CSF1 to predispose lung fibrosis in humans.

## Supplementary Material

1
