# Supplementary material for: Constitutive AKT Activity Predisposes Lung Fibrosis by Regulating Macrophage, Myofibroblast and Fibrocyte Recruitment and Changes in Autophagy
Source: Adv Biosci Biotechnol. Author manuscript; Available in PMC 2019 Nov 20. (PMC6866236; doi:10.4236/abb.2019.1010027)
Supplement: 1 [file NIHMS1058098-supplement-1.pdf]

Supplemental Material

Supplemental Table 1. qRT-PCR primers.

Murine primers for qRT-PCR: (5' to 3')		
Il-12	(forward)	ggaagcacggcagcagaata
	(reverse)	aacttgagggagaagtaggaatgg
Inos	(forward)	accctaagagtcaccaaaatggc
	(reverse)	ttgatcctcacatactgtggacg
Il-10)	(forward)	gccaagccttatcggaaatg
	(reverse)	tttctgggccatgcttctct
Ym1	(forward)	ctggaattggtgcccctaca
	(reverse)	caagcatggtggttttacagga
ColIa	(forward)	atggattcccgttcgagtacg
	(reverse)	tcagctggatagcgacatcg
ColIIIb	(forward)	caccettcttcatcccactctta
	(reverse)	accaaggtggctgcatcc
aSMA	(forward)	gctcagccagatgcaatcaa
	(reverse)	ccttggccacaatggtcttg
Beclin1	(forward)	caatgtcttcaatgccacctt
	(reverse)	ttcattccactccacaggaac
Map1lc3a	(forward)	gcctgtcctggataagacca
	(reverse)	ggttgaccagcaggaagaag
Map1lc3b	(forward)	cgtcctggacaagaccaagt
	(reverse)	ccattcaccaggaggaagaa

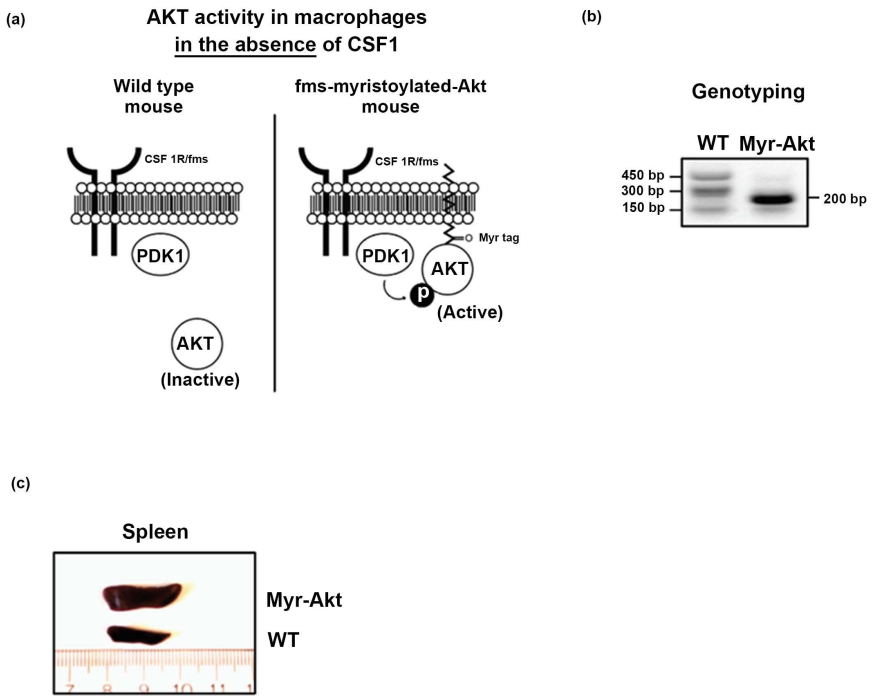

Supplemental Figure 1. Schema for constitutive activation of AKT and identification of Myr-Akt mice. (a) AKT is active in macrophages in the absence of CSF1 in Myr-Akt mice. (b) Genotyping identifies the 200 bp band that distinguishes Myr-Akt from WT mice. (c) Splenomegaly as confirmation for Myr-Akt penetrance.

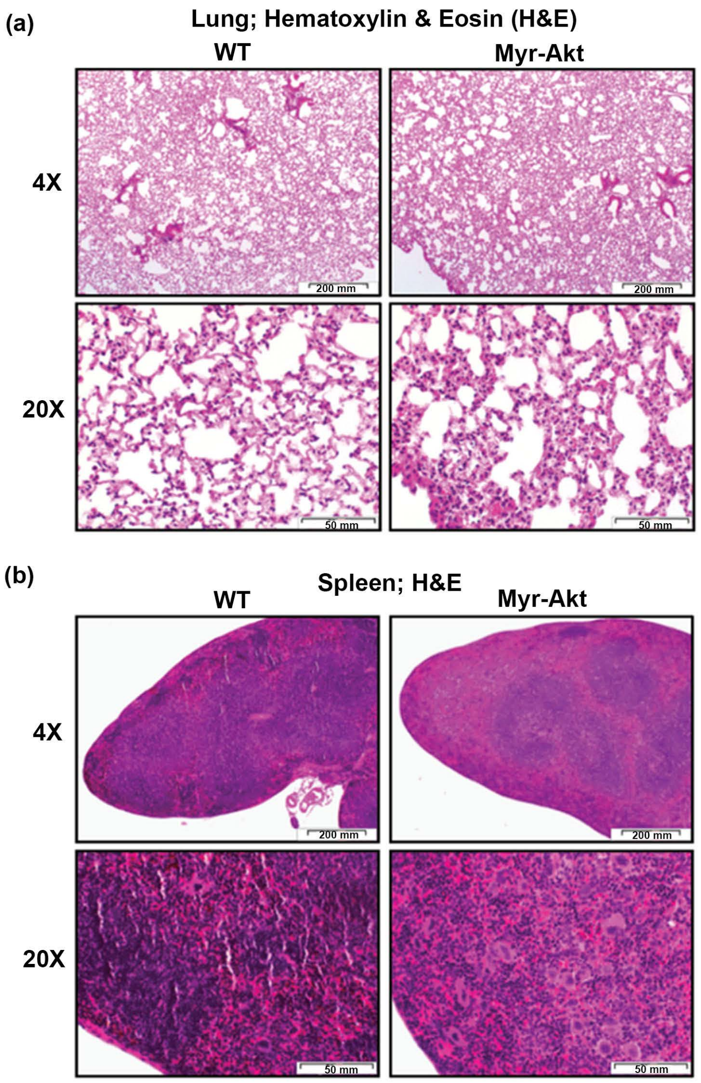

Supplemental Figure 2. Tissue architecture in Myr-Akt mice. 6 - 8-week-old WT or Myr-Akt mice were sacrificed and (a) lungs and (b) spleens harvested, paraffin embedded, and subjected to Hematoxylin & Eosin (H&E) staining and microscopy using 4X and 20X objectives (40× and 200× magnification, respectively).

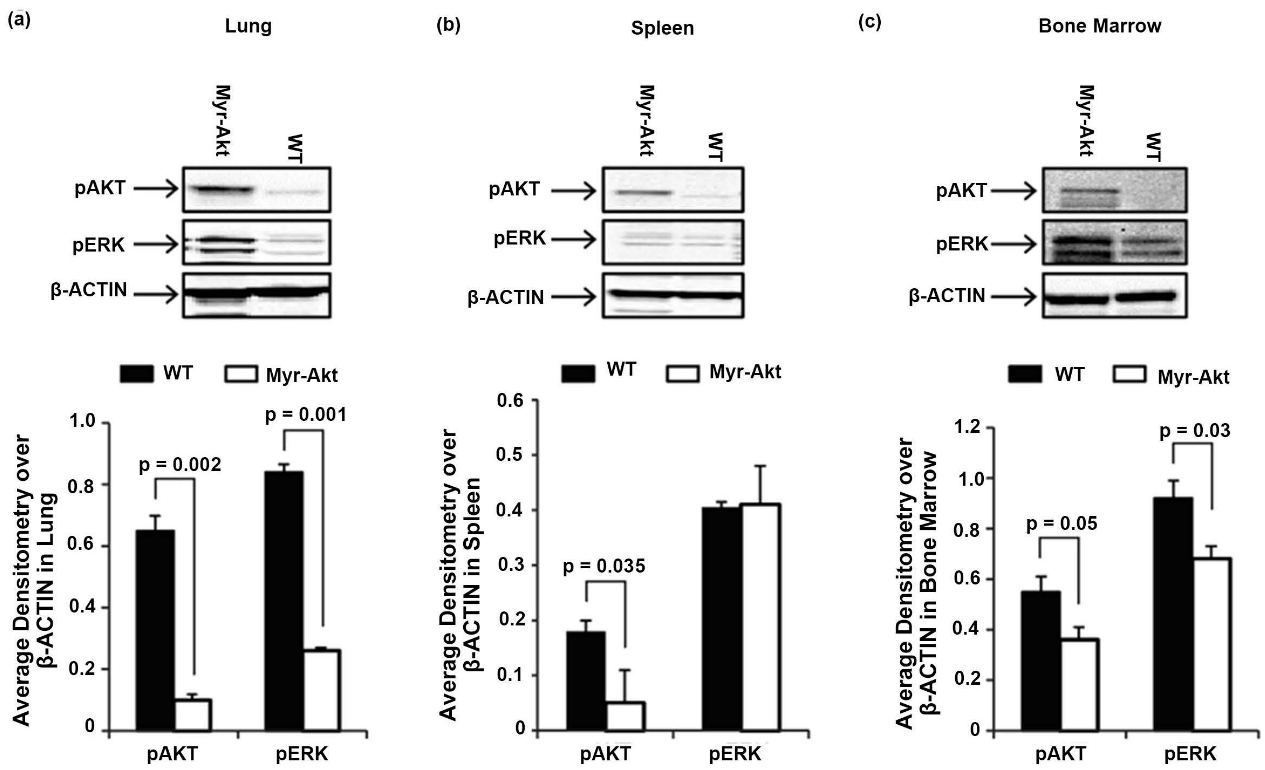

Supplemental Figure 3. Signaling pathway activation in various tissues in Myr-Akt mice. 6 - 8-week-old WT or Myr-Akt mice were sacrificed and (a) lungs, (b) spleens, and (c) bone marrow were harvested and subjected to Western Blot analysis for differences in phosphor-Akt and phospho-EKR relative to β-ACTIN expression.
